# Supplementary figures and images for: Mapping Invasion Potential of the Pest from Central Asia, Trypophloeus klimeschi (Coleoptera: Curculionidae: Scolytinae), in the Shelter Forests of Northwest China
Source: Insects. 2021 Mar 12;12(3):242. doi: 10.3390/insects12030242 (PMC8000299; doi:10.3390/insects12030242)

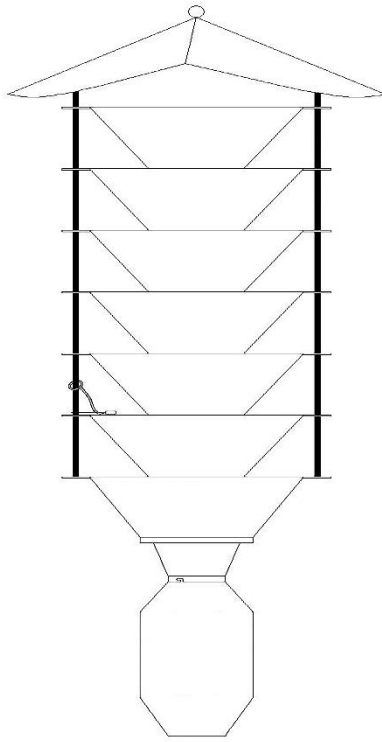

**Figure 1.** Schematic diagram of the trap.

Supplement: Supplementary file 1 [file insects-12-00242-s001.pdf]
